# The elusive role of herpesviruses in Alzheimer’s disease: current evidence and future directions

**DOI:** 10.1515/nipt-2023-0011

**Published:** 2023-07-10

**Authors:** Stacey L. Piotrowski, Allison Tucker, Steven Jacobson

**Affiliations:** Viral Immunology Section, National Institute of Neurological Disorders and Stroke, National Institutes of Health, Bethesda, MD, USA; Comparative Biomedical Scientist Training Program, National Institutes of Health, Bethesda, MD, USA; Department of Comparative Pathobiology, College of Veterinary Medicine, Purdue University, West Lafayette, IN, USA

**Keywords:** Alzheimer’s disease, Epstein–Barr virus, herpes simplex virus type 1, herpesvirus, human herpesvirus 6, infectious hypothesis

## Abstract

Alzheimer’s disease (AD) is the most common cause of dementia. While pathologic hallmarks, such as extracellular beta-amyloid plaques, are well-characterized in affected individuals, the pathogenesis that causes plaque formation and eventual cognitive decline is not well understood. A recent resurgence of the decades-old “infectious hypothesis” has garnered increased attention on the potential role that microbes may play in AD. In this theory, it is thought that pathogens such as viruses may act as seeds for beta-amyloid aggregation, ultimately leading to plaques. Interest in the infectious hypothesis has also spurred further investigation into additional characteristics of viral infection that may play a role in AD progression, such as neuroinflammation, latency, and viral DNA integration. While a flurry of research in this area has been recently published, with herpesviruses being of particular interest, the role of pathogens in AD remains controversial. In this review, the insights gained thus far into the possible role of herpesviruses in AD are summarized. The challenges and potential future directions of herpesvirus research in AD and dementia are also discussed.

## Introduction

Alzheimer’s disease (AD) is the leading cause of dementia, with an estimated 50 million people affected worldwide [1–3]. AD is a multifactorial neurodegenerative disease, where characteristics such as genes and environment are believed to play important roles [4, 5]. A pathologic hallmark of AD is extracellular senile plaques, which are comprised of beta-amyloid (Aβ) and accumulate in regions of the brain such as the hippocampus [5, 6]. While these pathologic changes are well-characterized, the pathogenesis leading to Aβ plaque formation and ultimately cognitive decline in AD is poorly understood [2, 5, 7].

Multiple hypotheses have been investigated in efforts to better understand the pathogenesis of AD [5, 8, 9]. For decades, the amyloid cascade hypothesis has spurred extensive research with the idea that Aβ deposition is the initial event in AD [8, 10]. Aβ accumulation is suspected to lead to further downstream effects such as intracellular tau neurofibrillary tangle formation, neuroinflammation, neuronal cell loss, neurodegeneration, and cognitive decline [7, 11]. While the reasons for the continued failures of clinical therapeutic trials that target Aβ are likely complex, including the current lack of biomarkers needed to identify patients early in disease progression and lack of objective clinical trial endpoint measures, it suggests that AD pathogenesis is likely not simply a function of the amyloid cascade [12–15]. Recently, interest in the role of infections and infectious triggers or contributors in AD has garnered renewed attention [16].

The idea that a pathogen may play a role in AD dates back to the early 1900s, where it was originally proposed by Alois Alzheimer and Oskar Fischer [17, 18]. Since then, numerous bacteria, fungi, parasites, and viruses have been investigated for an association with AD [17, 19]. While a variety of viruses have been investigated for their potential role in AD, including human immunodeficiency virus (HIV), hepatitis C virus (HCV), and the recent severe acute respiratory syndrome coronavirus 2 (SARS-CoV-2, COVID-19), herpesviruses have been a consistent focus of attention in the AD field (Table 1) [17, 19–25]. However, evidence for the role of herpesviruses in AD, and the infectious hypothesis in general, remains highly debated [26, 27].

**Table 1:** Timeline of selected publications investigating the role of viruses in Alzheimer’s disease (AD).

Pathogen	Year of Publications
Herpes simplex virus type 1 (HSV-1)	1982 [67]
	1997 [82]
	2008 [79]
	2009 [68,78,155]
	2011 [73]
	2018 [69]
	2019 [70, 71, 75]
	2020 [72, 76, 81]
	2021 [74, 77, 83]
	2022 [84]
Human herpesvirus 6 (HHV-6)	2014 [104]
	2015 [102]
	2017 [103]
	2018 [46, 69]
	2020 [98]
Epstein–Barr virus (EBV)	2014 [104]
	2021 [110, 118]
Cytomegalovirus (CMV)	2018 [123]
Varicella zoster virus (VZV)	2018 [120]
	2021 [119, 121]
	2022 [122]
Hepatitis C virus (HCV)	2014 [24]
Human immunodeficiency virus (HIV)	2017 [23]
	2021 [25]
Severe acute respiratory syndrome coronavirus 2 (SARS-CoV-2, COVID-19)	2022 [21]

## Infectious hypothesis of AD

Neurons are likely the primary source of Aβ in the brain [28]. Aβ is generated via the cleavage of amyloid precursor protein (APP) by β-secretase, followed by γ-secretase [29, 30]. An imbalance of Aβ production and clearance is thought to contribute to the excessive accumulation of Aβ in AD [31]. While the accumulation of Aβ is potentially neurotoxic, evidence suggests that Aβ may initially be beneficial [32].

Aβ is an evolutionary conserved peptide, suggesting it serves an important physiological role [33, 34]. LL-37, the human antimicrobial peptide (AMP), is structurally similar to Aβ, with both proteins able to form oligomers and insoluble fibrils [35]. Evidence that Aβ functions as an innate AMP has recently grown, with both *in vitro* and *in vivo* studies showing that microbial infection can increase Aβ production and aggregation [36]. Additionally, *in vitro* assays have also demonstrated the antimicrobial activity of Aβ against a variety of pathogens [35, 36].

**Figure 1:**
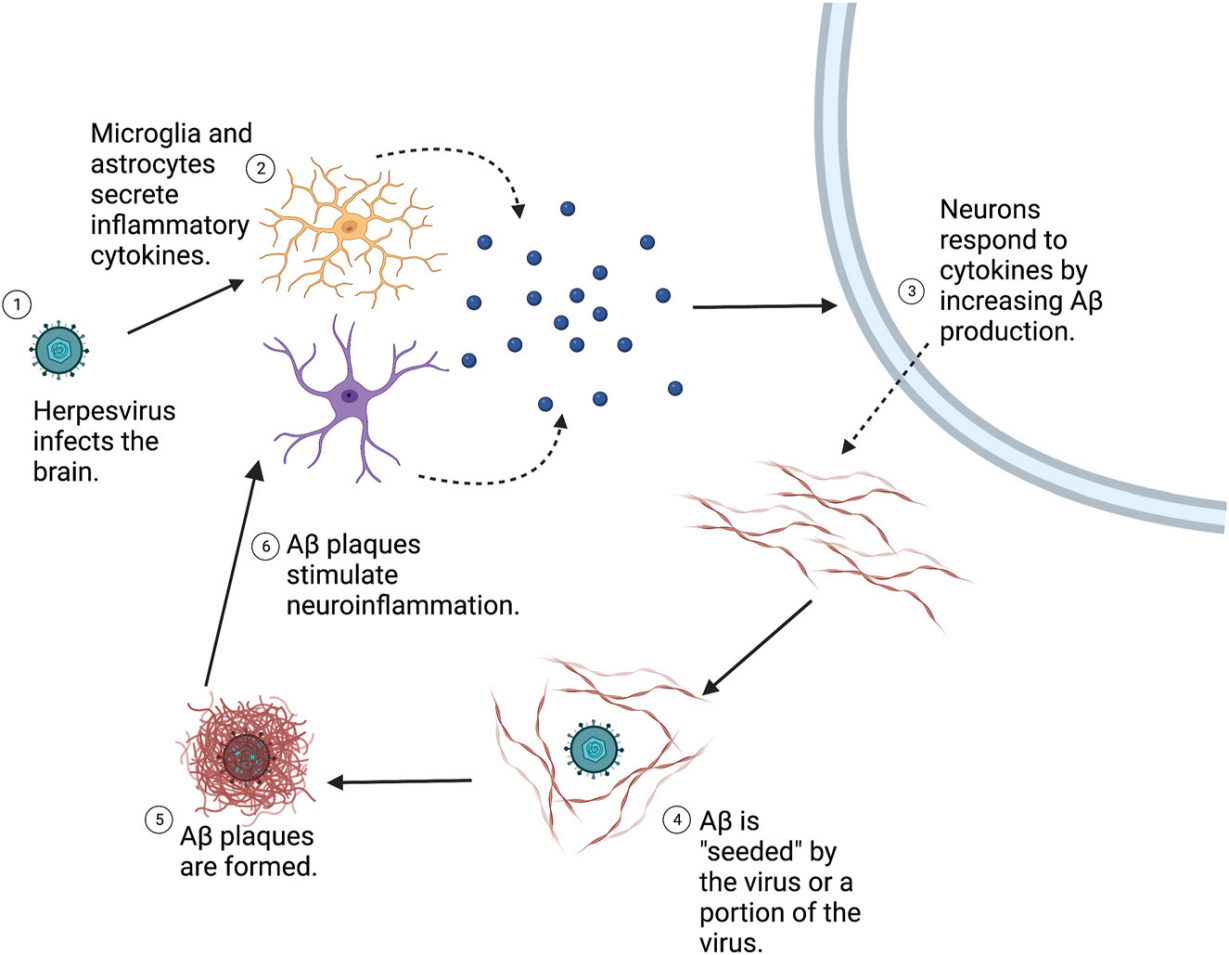
Herpesviruses and the infectious origin hypothesis of Alzheimer’s disease (AD). The infectious origin hypothesis of AD theorizes that the following general events may occur in response to pathogens that enter the brain, ultimately leading to beta-amyloid (Aβ) plaque formation. 1 The pathogen, such as a herpesvirus, infects the brain. 2 In response to the viral infection, microglia and astrocytes secrete pro-inflammatory cytokines, such as interferon. 3 Neurons respond to the binding of these cytokines by increasing Aβ production, via mechanisms like interferon-induced transmembrane protein 3 (IFITM3)-mediated γ-secretase activity. 4 Aβ is “seeded” and nucleated by the virus or a portion of the virus such as glycoproteins, with Aβ fibrils aggregating around the virus and trapping it. 5 Aβ plaques that are seen in AD are ultimately formed as Aβ continues to aggregate around the virus. 6 Aβ aggregation stimulates further microglial activation and neuroinflammation, further contributing to continued Aβ production. Figure 1 was created with BioRender.com.

One avenue of investigation in the infectious hypothesis theorizes that pathogens are the primary cause of AD, triggering the amyloid cascade and ultimately leading to plaque formation and other neurodegenerative changes associated with AD [16, 19]. Due to Aβ′s function as an AMP, it has been proposed that microbes, such as herpesviruses, that enter the brain can function as “seeds” for Aβ aggregation and fibrillation (Figure 1) [37, 38]. When viruses enter the brain, microglia and astrocytes are stimulated to produce cytokines such as interferon, which may increase Aβ production from neurons through interferon-induced transmembrane protein 3 (IFITM3)-mediated γ-secretase activity [39]. Aβ is then nucleated by the pathogen or a portion of the pathogen, such as glycoproteins in the case of viruses, triggering fibril aggregation and organization around the invading infectious agent, subsequently protecting the brain from further infection but ultimately causing the Aβ accumulation and plaque formation seen in AD [18].

In addition to the potential role of microbes as the “infectious origin of AD,” there has been growing interest in the possible contributions of viral infection to AD progression, the “infectious comorbidity in AD” theory [40]. As in other chronic neurodegenerative diseases, the idea of “two hits” or “multiple hits” contributing to disease manifestation, including genetic predispositions, oxidative stress, neuroinflammation and environmental factors such as pathogen exposures, has gained traction in AD research [41–43]. In addition to the direct “seeding” of plaque formation by viruses as a potential causative element, the multifactorial nature of AD suggests that viruses and other infectious agents may act as contributory factors in some subtypes of AD patients through a variety of mechanisms [4, 44].

Some viruses have a unique ability to integrate into human host cell chromosomes as a part of their life cycle or incidentally, potentially allowing for disruptions in gene expression and other effects for the host cell that may contribute to the development of disease [45, 46]. Some pathogens, such as herpesviruses, result in lifelong latent infection that may cause intermittent damage that contributes to AD progression after reactivation [40, 47, 48]. Both acute and chronic viral infection can activate microglia and stimulate cytokine release, with resulting neuroinflammation that may drive and influence both Aβ and tau pathology [49–52]. Aβ aggregation has been shown to subsequently further induce microglial activation and stimulate inflammatory mediators, creating a cycle of enhanced protein deposition and continued neuroinflammation [37, 53, 54]. Microglial activation has also been shown to activate A1 astrocytes and contribute to a neurotoxic response, resulting in the death of neurons and oligodendrocytes [52, 55]. Neuroinflammation, which can be mediated by viral infections, may ultimately drive neurodegeneration and disease progression in AD [56].

Additional mechanisms by which viruses may contribute to AD are also being explored. Dysregulated autophagy, which is associated with the progressive pathology of AD, has also been associated with neurotropic viruses [57]. While microRNAs (miRNAs) have been investigated as a potential biomarker of AD, viral infection may also contribute to the dysregulation of miRNA activity that has been linked to AD [58, 59].

Due to their ubiquitous nature, their association with other neurodegenerative diseases, and their life cycle characteristics, herpesviruses have been intensely investigated and scrutinized for their potential role in AD [18, 26, 60, 61]. This interest has spanned across multiple decades and has increasingly focused on specific herpesviruses in recent years, with most attention concentrated on herpes simplex virus type 1 (HSV-1), human herpesvirus 6 (HHV-6), and Epstein–Barr virus (EBV). Varying associations in a multitude of studies highlight the need for additional research and further scrutiny of the possible role of herpesviruses in AD pathogenesis.

## Herpes simplex virus type 1 (HSV-1)

Herpes simplex virus type 1 (HSV-1), a neurotropic virus that can establish a latent infection with subsequent reactivation, has been investigated for decades as a possible factor in AD (Table 2) [62, 63]. Infection usually occurs in childhood, with approximately 70 % of the worldwide population having antibodies to HSV-1 [64]. HSV-1 infection can result in a wide-variety of clinical manifestations, including infection of the central nervous system and encephalitis [65, 66].

**Table 2:** Selected publications investigating the possible role of herpes simplex virus type 1 (HSV-1) in Alzheimer’s disease (AD).

Reference	Results	Methodology
Wozniak et al. [68].	Increased HSV-1 DNA co-localization with Aβ plaques in AD patients	*In situ* PCR and immunohistochemistry
Eimer et al. [69].	Aβ fibrillization and oligomerization mediated by HSV-1 Aβ protects against HSV-1 infection	*In vitro* culture 5XFAD transgenic and wild-type mice
Linard et al. [81].	*APOE4* carriers with IgM or high IgG for HSV-1 had increased risk for AD	Prospective cohort and chemiluminescence immunoassay
Bocharova et al. [74].	Aβ does not protect against HSV-1 infection	5XFAD transgenic and wild-type mice
Murphy et al. [83].	HSV-1 seropositivity associated with cognitive decline, not dementia and AD	Prospective population-based cohort and chemiluminescence immunoassay
Zhang et al. [84]	Anti-HSV-1 IgG not associated with risk of AD	Multigenomics analysis and two-sample Mendelian randomization analysis

In 1982, reactivation of HSV-1 infection was initially suggested as a possible cause of neurodegeneration and AD due to similarly affected regions of the brain [67]. HSV-1 DNA was later shown to co-localize with Aβ plaques to a greater extent in AD patients than age-matched controls, suggesting that HSV-1 may be the cause of AD-related plaque formation [68]. More recently, *in vitro* assays showed Aβ fibrillization and oligomerization mediated by HSV-1 [69]. The interaction between the viral protein corona of HSV-1 and the extracellular environment has been demonstrated as a critical component of HSV-1 induced Aβ accumulation [70]. A 3D human brain-like tissue model comprised of human-induced neural stem cells also demonstrated extensive Aβ fibrillary plaque-like formations upon lower dose inoculation of HSV-1, coupled with an upregulation in presenilin 1 (PSEN1) and presenilin 2 (PSEN2), the catalytic subunits of γ-secretase, and an increase in glial activity and markers of neuroinflammation [71]. Additionally, HSV-1 has been shown to depolarize the plasma membrane of mouse cortical neurons and increase intracellular calcium, which initiates APP production similar to a transgenic mouse model of AD [72, 73]. However, *in vivo* models have led to varying results, with one study showing protection against HSV-1 encephalitis in 5XFAD transgenic mice that express human Aβ, while another study with a lower dose of viral inoculum failed to show Aβ protection from HSV-1 infection in the same transgenic strain, coupled with a lack of evidence of HSV-1-triggered Aβ aggregation [69, 74].

Repeated infection of HSV-1 has been associated with AD-like neurodegeneration. In transgenic mice, non-Aβ AD biomarkers, such as hyperphosphorylated tau protein and astrogliosis, increased with recurrent HSV-1 infection [75]. The impact of HSV-1 infection on the hippocampus includes the accumulation of AD biomarkers in the dentate gyrus (DG) alongside increased neuroinflammation and lymphocytic infiltration in mouse models [75–77]. In both *in vitro* and *in vivo* models, proliferation and differentiation of neural stem cells (NSCs) in the DG decreased post-HSV-1 infection [75–77]. Changes in gene expression contribute to immunological cascades associated with AD pathogenesis and increase interferon-gamma, with some of these cascades linked to the upregulation of miRNA-146a [78, 79]. miRNAs expressed by HSV-1 can dysregulate host miRNA activity; however, these changes are often restricted to cell type or environment [80].

Studies investigating HSV-1 as a risk factor for AD have suggested that individuals with both HSV-1 positivity in the brain and apolipoprotein E4 (APOE4) allele, a genetic susceptibility factor for AD, have an increased risk for AD [81, 82]. However, a recent population-based study did not find an association between HSV-1 seropositivity and risk of dementia, but seropositivity was associated with subtle cognitive decline [83]. Additionally, a multigenomics analysis and two-sample Mendelian randomization study found no association between anti-HSV-1 immunoglobulins and risk of AD [84]. The often-disparate results between studies emphasizes the need for continued investigation using a multitude of methodologies to more completely characterize the potential role of HSV-1 in AD.

## Human herpesvirus 6 (HHV-6)

Over 95 % of people over the age of 2 are seropositive for HHV-6, a betaherpesvirus [85]. HHV-6A and HHV-6B are two distinct variants, with evidence suggesting that HHV-6A is more neurotropic [86]. While primary childhood infection with HHV-6B can be asymptomatic or the cause of febrile illness, HHV-6A and -6B have both been associated with neurological diseases such as mesial temporal lobe epilepsy, encephalitis, and multiple sclerosis (MS) [87, 88]. Interestingly, the brain regions impacted by HHV-6 infection in diseases such as mesial temporal lobe epilepsy can be co-localized to regions of interest in AD, like the hippocampus [89–91].

The notion that HHV-6 could be associated with Alzheimer’s disease was strongly ignited by two recent publications (Table 3) [46, 69]. *In vitro* assays showed Aβ fibrillization and oligomerization mediated by HHV-6 and its viral glycoproteins [69]. Using advanced multiomics analyses, increased HHV-6A and human herpesvirus 7 (HHV-7) were found in brains of individuals with Alzheimer’s disease [46]. Dysregulation of miRNA155 was identified as a potential contributor to neuroinflammation in AD, with additional studies showing that HHV-6A infected immune cells actively suppress miRNA155 activity [46, 92, 93]. While intriguing and exciting, these bioinformatics analyses and their implications have been questioned, spurring further analyses and varying interpretations based on the computational pipelines utilized on the omics data [94–97].

**Table 3:** Selected publications investigating the possible role of human herpesvirus 6 (HHV-6) in Alzheimer’s disease (AD).

Reference	Results	Methodology
Carbone et al. [104].	Increased HHV-6 positivity in peripheral blood leukocytes (PBL) in AD patients; PBL positivity increased in patients who developed AD	Nested and/or quantitative real-time PCR (qPCR)
Agostini et al. [102].	No correlation between HHV-6 IgG and AD	Enzyme-linked immunoassay (ELISA)
Westman et al. [103].	Decreased HHV-6 IgG in AD	Multiplex immunoassay
Eimer et al. [69].	Aβ fibrillization and oligomerization mediated by HHV-6	*In vitro* culture
Readhead et al. [46].	Increased HHV-6A in brains of individuals with AD	Multiomic and computational analyses
Allnutt et al. [98].	No association between HHV-6 and AD	RNA-seq analysis and digital droplet PCR (ddPCR)

Despite using many of the same cohorts, a subsequent study failed to support an association between HHV-6 and AD when HHV-6 PCR reactivity of DNA extracted from AD and control brains was quantified, with low frequency of HHV-6 detection and low magnitude of HHV-6 PCR reactivity in cohorts [98]. In this study, the Broad Institute tool PathSeq was also used to screen the RNA-seq data for over 25,000 microbes including multiple herpesviruses, and it demonstrated no difference in viral detection between AD patients and controls [98]. A similar RNA-seq analysis in a different cohort also found similar HHV-6 abundance in AD and control brains [99]. Other transcriptomic profile comparisons revealed shared gene expression between AD patients and those with active HHV-6 infection [100, 101]. These seemingly disparate results have fueled discussions on the varying methodologies and analyses utilized in bioinformatics and omics-based approaches, their sensitivities for viral detection when compared to other more traditional assays such as PCR, and the different results anticipated from direct methods, such as PCR, and indirect methods, including serology [46, 94–98].

Other studies investigating the association between HHV-6 and AD via immunological measures have also achieved divergent results. While one study found decreased HHV-6 IgG in AD patients, suggesting a possible impaired humoral immunity to HHV-6 in AD, another study failed to find any association between the humoral response against HHV-6 and AD [102, 103]. A study in Italy found increased HHV-6 positivity in peripheral blood leukocytes of AD patients, which was also associated with clinical AD progression and suggested possible peripheral reactivation of HHV-6 may play a role in AD patients [104]. However, this relationship between leukocyte positivity in AD patients was not present in a similar study utilizing a different cohort [103].

Changes in AD associated pathology have been observed in cell cultures post-exposure to HHV-6A, with microglia infected with HHV-6A increasing Aβ1-42 expression [105]. Additionally, HHV-6A infection of peripheral blood mononuclear-microglial cells resulted in an increase in both tau and hyperphosphorylated tau [105]. While mice are not naturally competent to HHV-6 infection, the closely related murine roseolovirus failed to increase cortical Aβ deposition in the transgenic 5XFAD mouse model [99]. However, like most studies in this field, these results don’t refute the infectious hypothesis of AD or the antimicrobial protection hypothesis of Aβ [99].

## Epstein–Barr virus (EBV)

Human herpesvirus 4, more commonly known as Epstein–Barr virus (EBV), has long been investigated as a contributor to the demyelinating disease MS, with recent publications lending further credence to its role in MS pathogenesis [88, 106], [107], [108]. Approximately 90 % of the worldwide population is estimated to be infected with EBV, with clinical syndromes varying in part due to age of initial infection [109]. Few studies have investigated the role of EBV in AD, with one study in 2014 suggesting that EBV, in addition to the previously mentioned HHV-6, may be a risk factor for AD, and it may contribute to AD progression based on PCR positivity for EBV in peripheral blood leukocytes from AD patients [104] (Table 4). A recently published Mendelian randomization study observed an association between mononucleosis, the result of primary EBV infection later in life, and AD, suggesting that further investigation of EBV and its possible role in AD pathogenesis is warranted and that antiviral therapies may be beneficial in AD prevention or treatment [110].

**Table 4:** Selected publications investigating the possible role of Epstein–Barr virus (EBV) in Alzheimer’s disease (AD).

Reference	Results	Methodology
Carbone et al. [104].	PBL positivity for EBV increased in patients who developed AD IgG levels for EBV increased in patients who developed AD	Nested and/or quantitative real-time PCR (qPCR) Enzyme-linked immunoassay (ELISA)
Huang et al. [110].	Significant association between mononucleosis and risk of AD	Two-sample Mendelian randomization study
Tiwari et al. [118].	EBV peptides can potentially form cytotoxic aggregates	*In silico* bioinformatics analysis

Based on cell cycle dysregulation mediated by EBV infection, it has been proposed that this dysregulation could also contribute to neurodegeneration in AD [111]. EBV may contribute to neuroinflammation in AD through the infection of monocytes and peripheral blood mononuclear cells [112–115]. Two known EBV antigens have been associated with an adaptive immune response in the cerebrospinal fluid of AD patients, with their presence triggering a cytotoxic immune cascade mediated by the proinflammatory activity of CD8+ T cells [116, 117]. *In silico* analysis of EBV proteins have revealed potential peptides capable of forming cytotoxic aggregates, with further *in vitro* and *in vivo* experiments needed [118].

## Other herpesviruses

Other herpesviruses have been implicated for their potential role in AD due to hypothesized interactions with other viruses [17]. Varicella zoster virus (VZV, human herpesvirus 3) has been found at an increased prevalence in AD patients, and antiviral therapy after VZV diagnosis has been associated with a reduced risk of dementia [119–121]. However, *in vitro* studies suggest VZV does not directly trigger Aβ pathology, but rather, it can reactivate HSV-1 and indirectly contribute to Aβ production [122]. Similarly, the presence of both cytomegalovirus (CMV) and HSV-1 antibodies, not either virus alone, has been described as a significant risk factor for AD, suggesting interaction of multiple herpesviruses may be key to AD [123].

## The challenges of demonstrating the potential role of herpesviruses in AD

Obtaining definitive evidence for the role of herpesviruses in AD has thus far remained elusive, highlighting the inherent difficulties in this area of investigation. The association of herpesviruses with AD does not prove causation, but it can and should stimulate further investigation, as in the case of the infectious hypothesis and AD [124–126]. A recent publication describes the evidence that may suggest a causal role for microbes like HHV-6 in disease, including, among others, viral nucleic acid or antibody levels that correlate to disease severity, lack of detection of other infectious agents in the diseased tissue, and specific antiviral therapy that decreases viral load and is followed by clinical improvement [27]. However, whether all of these criteria are widely-applicable to proving the role of herpesvirus infection in AD is unknown, particularly as some criteria, such as presence of viral nucleic acids, does not immediately allow for characterization of a virus as a cause, cofactor, or bystander in the disease process [27]. Additionally, since the infectious hypothesis suggests that many infectious agents may play a role in AD, antimicrobials directed at one specific agent may not be widely successful or beneficial if it is not combined with more general therapeutics aimed at other contributors to AD such as neuroinflammation [27, 36, 127]. As the necessary specific criteria and threshold to prove causation remains undefined, evidence for the role of herpesviruses in AD can only grow with continued investigation and with replication of results through various methodologies and in multiple different cohorts [27].

The lifecycle of herpesviruses provides additional obstacles for proving their potential role in AD. Herpesviruses cause a lifelong infection, with initial infection usually occurring at an early age [27]. If herpesviruses play a role in AD, the interval from primary infection to clinical disease onset is decades. Current analytical methods, including omics-related analyses and more traditional diagnostic assays such as PCR, may not be specific or sensitive enough to detect the remnants of viral infection that may be present in AD tissue decades later [18, 27]. Additionally, the host-herpesvirus interaction allows for two different life cycles, latent and lytic cycles, where primary infection and reactivation could potentially play different roles in disease pathogenesis [19, 60, 128]. More recently developed investigative strategies and techniques, such as extracellular vesicle interrogation, and novel analytical methods may be necessary to help elucidate further evidence of herpesvirus infection and its role in AD patients [129–132]. Extracellular vesicles, membrane-bound structures that contain a wide-variety of cargo, may serve as sensitive and specific biomarkers for viral infection and AD, while also serving as a potential treatment modality to deliver therapeutic cargo [133].

Herpesviruses are also ubiquitous, with all humans infected with at least one herpesvirus in their lifetime [128]. A ubiquitous pathogen like HHV-6 can cause a specific disease in a subset of infected individuals, as many factors, such as genetics and comorbidities, may contribute and ultimately culminate in disease manifestation [27]. AD is multifactorial and heterogenous, with factors like genetics, inflammation, and environmental aspects contributing to various extents in different subgroups of patients [4]. While they may not be the sole cause of AD, herpesviruses could be a contributing factor in some, but not all, AD patients [44].

Multiple *in vitro* and *in vivo* studies have shown that Aβ can act as an antimicrobial peptide against a variety of pathogens, and Aβ plaque formation can also be seeded by many microbes [35, 36, 134, 135]. Some meta-analyses have associated a variety of herpesviruses with an increased risk of AD, highlighting a possible lack of specificity for a single herpesvirus as a cause of AD [136, 137]. Multi-pathogen infections, including both single-taxon and multi-taxon combinations, and overall infectious burden may be greater contributors to AD, and the relevant combinations of beneficial antimicrobial therapeutics may differ from patient to patient [138–141]. Studies that focus on a single microbe in AD, such as HHV-6 or HSV-1 as a sole cause or contributory factor in AD, may erroneously simplify the clinical scenario where multiple infectious agents, in combination with other attributes such as genetics, contribute to disease manifestation and progression. While studies analyzing multiple contributory agents or other components may be more difficult to design and interpret, they are likely necessary to more fully understand the effects of herpesvirus infection in AD. The proper methodology for determining which patients may have viral infection as a factor in their AD pathogenesis is not clear, but it is likely essential for the success of any forthcoming antiviral-based AD treatment clinical trials. The establishment of pathogen-based biomarkers may aid in identifying patients with viral contributors to AD and help distinguish appropriate trial and treatment populations [56].

## Potential future directions of herpesvirus research in AD

Failure of Aβ-targeting therapeutics has recently called into question whether Aβ is the major contributor to AD progression [11]. The challenges of successfully targeting Aβ, such as determining the appropriate timeframe and adequate dosages for therapeutic administration, have also led to the diversification of targets for AD treatments [12], [13], [14], [15, 142, 143]. In addition to Aβ plaques, neurofibrillary tangles, intraneuronal aggregates of hyperphosphorylated and misfolded tau protein, are also a pathologic hallmark of AD [144, 145]. While accumulation of each protein is likely vital to AD initiation and progression, evidence suggests that the interplay between Aβ and tau is complex and likely drives neuronal disease [146]. For example, while Aβ has been shown to trigger pathologic tau modifications, other evidence suggests that tau also enhances the toxicity of Aβ [146–150]. Recent studies have more closely associated tau pathology to cognitive decline in AD [151–153]. This has brought increased attention to the tau hypothesis, which theorizes that tau is the initiator of downstream AD neurodegeneration via pathologic modifications, like hyperphosphorylation, that make the protein prone to aggregation and prion-like propagation [7, 8, 154]. However, if tau plays a primary role in AD pathogenesis, that does not preclude infectious agents, like herpesviruses, from contributing to AD. The few studies investigating the effects of viral infection on tau have shown a transient tau increase in neurons after HSV-1 infection and HSV-1 induced phosphorylation [155–157]. Additionally, viral glycoproteins may promote release of extracellular vesicles and the transfer of misfolded tau proteins between cells [129]. As the tau hypothesis continues to be placed near the forefront of theories of AD pathogenesis, further research on the effects of viral infection on tau aggregation and propagation is warranted, as infectious agents may affect the accumulation of multiple neurodegenerative proteins involved in AD.

Alpha-synuclein (α-syn) is a synapse-associated protein that can abnormally accumulate, forming Lewy bodies, and is linked to a group of neurodegenerative diseases called synucleinopathies, which includes Parkinson’s disease, multiple system atrophy, and dementia with Lewy bodies [158–161]. Multiple studies have demonstrated that in addition to Aβ and tau, α-syn pathology can also be found in over 50 % of AD patients, suggesting that α-syn may also be involved in AD pathogenesis or that many AD patients have other neurodegenerative disease co-morbidities [162–165]. Few studies thus far have investigated the relationship between viral infection and α-syn; however, those studies suggest a possible role for α-syn as an inhibitor of viral infection, similar to Aβ [166–169]. The effects of herpesvirus infection on α-syn aggregation in AD patients and other neurodegenerative diseases has not yet been investigated, but this research is likely necessary for a deeper understanding of the role of microbes in AD and dementia pathogenesis.

*In vivo* studies can be useful in the investigation of the effects of herpesviruses in AD, as the capacity of viruses to influence disease pathology in a more complete animal model system can provide important evidence of causation [27]. Transgenic mouse models that result in Aβ plaque and tau tangle formation are the most common animal models of AD [170, 171]. However, many experimental treatments that are successful in pre-clinical studies utilizing rodent models fail in clinical human trials, which suggests that animal models may not be good surrogates of human AD or better animal models that more closely mimic human clinical AD are needed for better result translation [170, 172, 173]. While most of the *in vivo* research on the role of herpesviruses in AD has utilized rodent models, other animal models may better replicate the effects of viral infection on Aβ and related protein accumulation in AD. Both spontaneous and induced models of AD have been described in nonhuman primates [174]. While these models also have some shortcomings when compared to human AD, they may provide an essential link between pre-clinical studies and clinical trial results due to their phylogenetic relationship to humans and their similarities in aging pathologies [174, 175]. Non-human primates can be infected with human herpesviruses of interest in AD research, such as HHV-6A and HHV-6B, and they also harbor closely related viruses such as rhesus lymphocryptovirus, Callitrichine herpesvirus 3, and Simian varicella virus [176–179]. Aged dogs also naturally develop AD-related pathology, including Aβ plaques, and a clinically similar syndrome called canine cognitive dysfunction [170, 180, 181]. The continued accumulation of a large number of longitudinal biosamples through the NIH-funded Canine Longevity Consortium and the Dog Aging Project provides a plethora of opportunities for investigating AD-related biomarkers and phenotypes in the canine model [182]. These longitudinal studies in an animal model relevant to human AD may provide additional insights into the association between risk factors like viral infection and cognitive decline. The utility of more translationally relevant animal models may strengthen the evidence for viral infection as a factor in AD.

The countless recent failures of later stage clinical trials targeting Aβ highlights the necessity for different therapeutic strategies in the treatment of AD [12, 13]. Based on the multifactorial nature of AD, polypharmaceutical strategies are likely to be most effective in treating AD [183]. If the infectious hypothesis of AD proves true, antiviral and antimicrobial agents, in combination with therapeutics targeting the overproduction of Aβ, may provide opportunities to dampen neuroinflammation secondary to microbial infection and reduce the Aβ burden [184]. A recent study based in Sweden showed that antiviral treatment was associated with a decreased risk of dementia, while herpesvirus infection without antiviral treatment resulted in an increased risk of dementia [121]. A population study in Taiwan revealed reduced risk of dementia in HSV-1 infected patients who received anti-herpetic medications [185]. A clinical trial of the antiviral valacyclovir in AD patients who are positive for HSV-1 or HSV-2 has been initiated [186]. A 4-week trial in early-stage AD patients determined a tolerable and safe high-dose treatment of valacyclovir; however, no significant change in biomarkers was noted in that time period [187]. The results of these initial clinical studies, coupled with continued *in vitro* and *in vivo* investigation of the role of herpesviruses in AD, will help dictate the future utility of antiviral strategies in AD treatment and prevention, and the fulfillment of arguably the most important criteria for herpesviruses as causative agents in AD: that elimination or reduction of the virus changes disease outcome [27, 188].

## Summary

Based on the current literature and evidence, the exact role of herpesviruses, and infectious agents in general, in AD is uncertain and not definitive. While it is possible that herpesviruses may play a role in some AD patients, whether they can function as an initiator of the disease cascade, drivers of disease progression, or both remains unclear. As the population of AD-afflicted individuals continues to grow and research-related support in the field increases, further investigation is warranted to better understand the contributions of microbes to AD pathogenesis. When the relationship between pathogens such as herpesviruses and AD becomes clearer, it will help dictate potential future avenues for disease prevention and treatment, such as antiherpetic therapeutics.
